# Philadelphia Hospital

**Published:** 1839-11-30

**Authors:** 


### CLINICAL LECTURES.

### PHILADELPHIA HOSPITAL.

#### HARE-LIP—CHRONIC GANGRENE.

*Saturday, November 23d*, 1839. Dr. GIBSON remarked:

The first case I show you, to-day, gentlemen, is one of hare-lip, complicated with cleft of the palate, occurring in a boy of eleven years of age. The term hare-lip, or labium leporinum, has arisen from the supposed resemblance the part bears to the upper lip of a hare or rabbit. We meet with two varieties of it, the single and double, of which the former is the most common, and consists of a fissure or perpendicular division of one or both lips, in some cases in the middle of the lip, but more generally on one side. In double hare-lip there are two fissures, having between them a little lobe or hanging portion. The disease is nearly always congenital, though it sometimes occurs from a wound. The upper lip is by far the most frequent seat of the disease. The edges of the fissure are rounded, and covered with the natural membrane of the lips.

Hare-lip, even in the most favourable form, occasions great deformity, and is a serious obstacle to the suckling of infants, while it very much interferes with the articulation of adults. The deformity is sometimes much increased by the incisor teeth projecting nearly horizontally. If the lower lip be affected, it will be found that the patient can neither retain his saliva, nor learn to speak without the greatest difficuly. You may readily imagine then how much this form of the disease, fortunately so rare, would affect the health and comfort of the patient.

It would be a most difficult undertaking, indeed, impossible, to account satisfactorily for this as well as other deformities occurring in the fœtus in utero. It is more important for us to attend to the remedial means in our power.

The only effectual treatment is an operation, and this should be done without delay. Many surgeons advise postponing the operation, in the case of children, until the fourth or fifth year, on the supposition that their cries and resistance would derange, entirely, the success of it; also from the dread of convulsions, to which they would be liable. Sir A. Cooper, in particular, mentions instances which have been reported to him, or have come under his immediate notice, where such operations have terminated fatally from an attack of convulsions,—he therefore has fixed upon the age of two years as the most advantageous period for the operation, dentition being completed about that time. My own opinion is similar, though I have known instances, and heard of others, in which children only three weeks old have been operated on without the smallest inconvenience.

The object of this operation is to bring the deformity to the state of a simple wound—by paring off the edges of the fissure, and then keeping them in contact until union takes place. To effect this, you will find a very convenient form to be the one I have always employed. Let the head of your patient be elevated, and securely held by an assistant. Having separated the internal membrane of the mouth and the frœnum, place between the lip and gum a narrow and flat piece of wood, some five or six inches long. Let this be held by another assistant. Now stretch the lip upon the wood, and beginning near the nostril, make an incision downwards, in a straight line, and remove the edge of the fissure. When you have done the same operation on the opposite edge—you will have a chasm left resembling the letter V inverted—you have now simply to draw the edges of the chasm together and retain them by the twisted suture, and in doing this recollect the importance of passing the pin first through the lower portion of the lip, in order that you may have it perfectly even. I have seen cases where, inadvertently, the operator has introduced the first pin at the upper portion of the lip, and on getting to the lower portion has discovered that one side of the lip was considerably lower than the other. Two or three pins will generally be enough. You complete the operation by passing a separate ligature around each pin, in the form of the figure 8, and then passing a roller around the whole. In the course of four or five days adhesion will have occurred, and you may remove the pins. When the incisor teeth project, as mentioned, they must be removed.

The same principles should govern the operation for double hare-lip, though it is rather more difficult to perform. Four incisions must be made instead of two, one on each side the middle projection, taking care to let one or two of the pins pass across the latter. There is generally but little hæmorrhage from this operation, and this is chiefly because the cut surfaces are so accurately applied to each other, but cases are recorded, and one in particular, by Louis, where a patient had bled to death in consequence of the pins not having been applied sufficiently deep, leaving the posterior surfaces of the incision separated. Remember, then, gentlemen, that the pins should be introduced at least two-thirds through the substance of the lip.

I have shown the way in which I perform this operation, and the kind of suture I prefer. Many excellent surgeons, however, and among them Sir A. Cooper, give preference to the common interrupted suture, on account of the difficulty which sometimes occurs in drawing out the pins, and the exposure thereby of the new adhesions to be broken up. It is true, that the threads of the common suture may be taken out more easily, but by waiting until we are certain of a firm adhesion having taken place, I have never had any trouble with the pins.

Hare-lip, as in the patient before you, is frequently complicated with fissure of the palate. This latter may occur as a simple division of the soft palate, or as a cleft in the palate bones, and in the palate processes of the superior maxillary bones. Like hare-lip, it not only affects, materially, the suckling of the infant, but in later life impedes deglutition, and changes the character of the voice, so as often to render its sounds unintelligible. Instances have occurred, also, of foreign bodies getting into the larynx and bronchiæ, where they have laid the foundation of fatal pulmonary affections. At other times great irritation and even ulceration have been produced by fluids and even solids being thrown from the mouth or stomach into the nares. Where this affection occurs together with hare-lip in an infant, the treatment used for the hare-lip will most generally effect a cure in the cleft of the palate—this is explicable from the continued, though slight pressure, that the bringing together the edges of the hare-lip fissure must exert on the two sides of the mouth. But with a patient like the one before us, this treatment would not be so apt to succeed, although I have no doubt that more or less benefit would result to him. You observe distinctly the wide separation in the roof of the mouth. The bones are at least half an inch apart, the fissure extending forward to meet the fissure in the lip, and backwards through the uvula, dividing it into halves; on the right side the front part of the superior maxillary bone is very prominent, and the teeth tolerably regular and natural. On the left, the same bone does not advance, but is rather below and behind the right. The teeth on this side, also, are carious and ill-conditioned, and the incisors stand out horizontally. The right nostril is perfect—the left communicates directly with the mouth. His articulation, you observe, is unintelligible, altogether constituting a very unfavourable case. The ordinary operation for the relief of fissure of the palate, is called staphyloraphy by Mr. Roux, who was, perhaps, the first surgeon who attempted it. In a memoir which he published in 1825, he shows that he succeeded in twelve cases, in re-uniting the edges of the soft palate. Since that time he has performed a great many operations with more or less success. The principles of the operation are simply to make raw the edges of the soft palate, and then to approximate them by means of ligatures, and produce union. This is not unattended with difficulty, and each surgeon who has followed Roux in the operation, has employed a different means for that purpose. Among the American surgeons who have been successful in this operation, Drs. Warren, Stevens, Mettauer and Hosack are, perhaps, the most prominent. With regard to the patient under consideration, I shall determine between to-day and next Saturday, whether it is likely that any benefit can result from an operation, and will then make known to you my decision.

The next patient is a man, in whom we have a specimen of that variety of gangrene which I have ventured to call chronic, and which is described by Mr. Pott as attacking particularly the toes and feet of old people. It appears, usually, without any previous inflammation, by a small bluish spot on the inside of one of the toes, from which the cuticle is soon detached. This spot spreads in every direction, sometimes slowly, and at others more rapidly, either attacking the toes one after another or simultaneously. The disease extends gradually to the foot and leg. The pain experienced is variable—many subjects are not aware of the existence of the disease until the toes are extensively affected, and others again suffer intensely in the ankle and foot, and especially at night, before any discolouration takes place. When the disease is fairly established, the effect on the constitution is soon shown,—restlessness, inability to sleep—oftentimes spasmodic twitching of the limb, and delirium, make their appearance. The fœtor from the part becomes extreme—almost insupportable to the patient himself; the soft parts are soon detached, and the bones drop off at the joints. Mr. Pott describes this form of mortification as occurring only in aged persons, but I have found it frequently among middle-aged subjects—and you observe in the case before you, that it has attacked a man of 28 or 30.

The causes of these affections are supposed to be internal, but they are not accurately known. An ossified state of the arteries has been frequently found accompanying it, but cannot be said to be in all instances the cause of it, as they have also been found perfectly healthy in fatal cases of the disease. Neither can it be said to proceed exclusively from obstructions in the vessels of the part, such as ligatures,—for we well know that the requisite supply of blood is furnished through collateral branches.

Mr. Pott's cases occurred principally in persons affected with gout; those which I have met with have been persons in the lower walks of life, who were badly fed and clothed, or who had been exposed to all kinds of weather, or who had undergone recent and long continued illness, which left them thin and weak. Unfortunately we know nothing of the history of the case before us. The man is from Wales, and it appears had been employed somewhere in this neighbourhood as a ditcher. He came into the house some six weeks ago, with acute meningitis, for which he was sent to the medical wards. The disease in his toes, seems to have begun there, and he was sent to our wards,—his brain still remaining in such a state, that nothing satisfactory can be elicited from him. He has never complained of the slightest pain or uneasiness in his toes. In feeling his arteries at the wrist there is no evidence of ossification, nor any at the anterior tibial. On the left foot, the small toe, and the next adjoining it, are affected. On the right, the large toe only. These toes are black, as far as their first phalangial articulation, and, in each one at this point, there is a red line, distinctly formed, which shows a disposition in the mortified part to drop off. The treatment that has been pursued at all times, in this affection, has been of little avail. Depletives and stimuli have by turn been taken up, and each method carefully watch ed. Bark, at one time, was very much depended upon—opium, too, possessed the confidence of many, and in particular of Mr. Pott, though I must say, that having given it repeated trials, I have never found much benefit from it. In carbonate of ammonia, and camphor, I have more confidence. Many execellent surgeons, rejecting all kinds of medicine, depend upon a mild and nutritious diet, for the cure of this affection.

With regard to the local applications in use, I believe the only benefit they produce is to relieve pain, and keep the parts clean, and free from fœtor. I may perhaps except blisters, which are very highly esteemed by some surgeons, though I myself have but in a single instance experienced benefit from their use. In this case, however, I am willing to try the effect of it conjoined with nutritive diet—though I fear it will be unsuccessful.* As a general rule, I should not recommend

* Dupuytren, supposing the disease to depend on arteritis, proscribed general and profuse bleeding, and by this means asserts that he relieved three-fourths of his patients; but Cruveilhier doubts the accuracy of this statement, and though believing in arteritis as the cause of the disease, prefers to deplete locally.

amputation. The disease has almost invariably returned into the stump and proved fatal.

---

ON PNEUMOTHORAX AND INTERMITTENT FEVER.

*No.* 2—*Winter Course.*

*Saturday*, *Nov.* 23.—Dr. GERHARD remarked: Pneumothorax is to be considered as a *lesion*, rather than a disease, since it is always secondary to a previously existing disorder, which, in the great majority of cases is phthisis pulmonalis. The subject of the present remarks is a negro man, who, for a year or more, has been labouring under a slight cough, and other symptoms of tubercular disease. One day, about a month since, while walking in the street, he was suddenly seized with oppressive dyspnœa, and pain in the right side of the chest. This sudden occurrence of pain and dyspnœa, is an important point in the diagnosis of pneumothorax: in pleurisy, they come on more gradually, and often after great exposure. The immediate cause of pneumothorax is frequently a strain or stretching of the pleura, in consequence of the distention of the lungs with air, and the pressure of the ribs upon them. A cavity was already formed near the surface of the lung, and breaks into the interior of the pleura. This would occur more frequently, were it not usually prevented by adhesions between the pleura, taking place around the abscess; so that it cannot break outwardly, and discharge itself into the cavity of the chest. When such adhesions do not take place, a communication is formed between the cavity of the pleura and the air passages, from the gradual approach of the contents of the abscess, until the pleura sloughs; and then perforation usually occurs suddenly. Symptoms of pleuritis follow the lesion, as pain, dyspnœa, &c. The occurrence of the inflammation is owing to the irritation produced by the action of the air on the serous membrane, and it takes place usually with great rapidity. In one case, which I observed, it was established in less than half an hour, and lymph was already deposited. The first pain which occurs is owing to the sudden rupture, and the consequent dyspnœa from the pressure of the air in the pleura upon the lung. Afterwards, the pain, and a portion of the dyspnœa, depend upon the succeeding inflammation, as well as the pressure of the air; and the symptoms pass gradually into those of chronic pleurisy.

In the present case, the dyspnœa is excessive; quite as great as in severe cases of chronic pleurisy, when the pleura is filled with pus and serum. The patient lies on the affected side, (the right,) so as to allow of the fullest expansion of the healthy lung. At each act of inspiration, the left shoulder, as you perceive, is elevated with a spasmodic effort. The sound yielded by percussion on the affected side is very clear, in consequence of the distention of the chest with air; this you all perceive. The voice has a metallic resonance; the respiration is amphoric, owing to the passage of air through the bronchi, into a large cavity lined with a hard membrane. The right side of the thorax is moderately enlarged.

When cases of pneumothorax come to a favourable termination, which is not common, the cure is produced by the effusion of lymph, which closes the opening of the pleura; and serum and pus take the place of air, so that the case becomes one of chronic pleurisy, and the remainder of its course is precisely that of the latter disease. In most cases it terminates fatally, either suddenly, from the excessive dyspnœa which immediately follows the rupture, or more gradually, from the effects of the secondary inflammation of the pleura, added to the previous disease, causing the perforation.

In the treatment of pneumothorax, we are to proceed upon the same general principles as in pleurisy, and trust to nature for the reparation of the lesion. In this case we have employed neither general nor local bleeding; but blisters to the affected side, accompanied by the internal administration of small doses of digitalis, combined with Dover's powder. The patient is to be kept perfectly quiet until the period of convalescence, when gentle exercise will be proper. The operation of paracentesis should never be performed, unless where there is imminent danger of death from the pressure of the air on the lung, or for the purpose of procuring the discharge of the pus, when the case becomes converted into chronic pleurisy. In ordinary cases, however, there is no necessity for resorting to the operation, and exposing still more the pleura to the external air.

Pneumothorax may occasionally arise from various diseases, producing abscess or sloughing of the lung and pleura, but in more than nine-tenths of the cases it is the result of tubercular disease of the lungs, in the manner already explained.

The well characterized case of pneumothorax, which was before us, induced me to make some remarks respecting its symptoms and history. The proper subject of the lecture is, however,

INTERMITTENT FEVER.

It is not the object of the present lecture to enter fully into the history of this disease, but to illustrate certain interesting points in the diagnosis, pathology, and treatment, by a reference to cases.

*Case* 1.—This man has been sick for about a week; but has been suffering with "dumb agues," of the quotidian type, from time to time, for the last three years. He has been several times free from the disease for intervals varying from one to five weeks. After having been well for three weeks, he was again attacked with intermittent on the 15th inst. For three days it was of the quotidian type, then became a tertian. The patient suffers no pain in the abdomen; the liver is not enlarged; spleen not felt below the ribs, but there is tenderness in the left hypochondriac region; flatness on percussion for an inch below the ribs, but not on the thorax itself; the spleen, therefore, appears to be only slightly enlarged. The complexion is rather florid, the patient being at present in the hot stage of the fever; but there is less paleness than usual in this disease, even in the apyrexia. The skin on the thorax is moist, but that of the hands and arms are dry; appetite good; pulse slightly excited; tongue moderately coated, but rather red.

The only treatment which the patient has received, is the administration of a purge: we shall order two grains of quinine, with one of rhubarb, to be taken six times during the first intermission; afterwards, gradually reduced to *twice.*

*Case* 2.—Two months since, this man had a quotidian intermittent, which was cured by the use of quinine. The disease having returned in the tertian form, he was admitted into the hospital. He has been treated with bark, Fowler's solution, quassia, and since the commencement of convalescence, with hydriodate of iron. At the period of his admission he had pains in the back,—was feeble, and has continued so ever since,—has become very pale and emaciated. His appetite was good until last week; there has been no soreness of the stomach, or vomiting. The arsenic produced no nausea, but the iron has caused it, to a slight degree. The change of countenance is striking and peculiar, as it mostly is in chronic intermittents; the paleness is extreme; the emaciation is considerable, but it did not commence so soon as the paleness. The tongue is whitish and moist, indicating only slight gastric disorder; there is a little soreness at the epigastrium and right hypochondrium; liver not materially enlarged; spleen greatly enlarged, and readily felt below the ribs; the sounds of the heart are of that clear, clacking sort, which accompany anemia; there are also palpitations, depending on the same cause. Fowler's solution was given in doses of ten drops, three times a day, without inconvenience. After the intermittent was overcome by its use, it was replaced by the hydriodate of iron.

*Case* 3.—This man was admitted on the 18th inst. The disease commenced as a tertian intermittent, and continued in this form for two months; it was checked by quinine for a time, but returned, and has since been irregular in its course, being sometimes tertian, sometimes quartan,—the latter is the form which it last assumed. The treatment, since his admission into the hospital, has consisted in the administration of one grain of quinine, every two hours, during the intermission. Quartan intermittents, contrary to the usual statements, we have found generally easy of cure,—the most difficult cases being of the quotidian type.

*Present condition of the patient.*—The face is pale; there is considerable œdema under the eyelids, which is owing to the predominance of serum in the blood. There is no œdema, however, in the feet, or any part of the body, though there probably will be, should the affections of the viscera become more confirmed. There is slight tension over the whole upper third of the abdomen; spleen enlarged, and plainly felt below the ribs; percussion there yields a flat sound, and the same for six inches above the edge of the ribs; liver somewhat enlarged, full and tense. There is no palpitation of the heart: the sounds of this organ have the clear character which we have already remarked as common in anemic conditions of the system.

*Case* 4.—This woman has had intermittent fever frequently before the present attack; she was sick with it throughout the fall, and a part of the summer, of last year. The present attack commenced in July; the type was at first quotidian. The paroxysms were suspended, at one time, for eight days, when they returned in the tertian form. The patient had been sick for three months before her admission into the hospital. After her admission, she was first treated with quinine in considerable doses, but it failed to produce any effect. We then gave her Fowler's solution, in doses of ten drops, three times a day, which produced giddiness and headach, nausea, and vomiting; the dose was then diminished. This remedy arrested the paroxysms four days ago, since which time it has been discontinued, and she has been again put on the use of quinine in small doses. The patient has been very pale and emaciated, but is improving rapidly in health and appearance.

*Case* 5.—This case is presented as an example of a disease very liable to be mistaken for intermittent fever, but widely differing from it. The man was admitted on the 19th inst., with many of the usual symptoms of intermittent. He is forty years of age; drinks moderately; is a weaver by trade. In the summer of 1838, he had an attack of dysentery; seven or eight years since, had frequent attacks of intermittent. The present disease commenced in October; he was seized with a chill while digging a cellar, and was obliged to go to bed. Since that time he has had chills every day, between 10 and 12 o'clock, A. M., but they have not recurred invariably at the same period. The chills have been followed by fever and profuse sweating, which lasted through the greater part of the night; the appetite has been bad,—bowels regular. A cough commenced a week or two after the chills, and has continued ever since, attended with irregular pains in the chest and shoulder. The expectoration consisted of whitish phlegm, without any mixture of blood. There has been some œdema of the feet, but not of the face.

*Nov. 20th.* The patient's complexion is sallow, with some emaciation; the functions of the brain are unimpaired; sleep good; countenance expressive of slight dyspnœa. Pulse feeble, and 116 in the minute; respiration 28. Tongue red, pointed, and rather dry. There is slight tenderness in the left hypochondrium and epigastrium, but the spleen cannot be felt below the ribs. There is thirst, the appetite is bad; bowels regular; cough short, with slight whitish expectoration; there is dyspnœa on ascending stairs, with palpitations, which have continued from the commencement of the illness. The respiration, *posteriorly*, is harsh on both sides; more vesicular and expansive in the upper part of the right than of the left; rude at the centre of the left lung; mucous rhonchus at its base. Percussion, posteriorly, yields a dull sound on the left side when compared with the right; but in no place a flat one. Respiration, anteriorly, is vesicular under the left clavicle, and throughout the whole anterior portion of the left side; action of the heart much quicker and more abrupt than usual,—not heaving; *bruit de soufflet* heard in the first sound; the second is short and clear, and somewhat exaggerated. Respiration on the right side vesicular.

*Nov.* 22*d.*—Had a chill this morning; was chilly through the whole night, but feet seemed warm to the attendants Dyspnœa continues with dilatation of nostrils. Pulse 108; respiration 32; tongue dry and reddish; abdomen rather large and slightly tympanitic, but without any eruption; cough as before; expectoration whitish, but more viscid, and streaked with blood; the relative force of the respiration is changed on both sides, and irregular. Respiration is now rude in the uper third of the left lung posteriorly; rude and feeble in the middle; tolerably vesicular below. On the right side posteriorly, it is vesicular in the upper half; very feeble in the lower half, and at times the voice is partially egophonic. Percussion, posteriorly, on the left side, returns a clear sound in the lower half; on the right side, at the lower part, flat, but gradually becoming natural towards the summit; no evident fulness of the side. Respiration, anteriorly, on the right side, very irregular; on the left, very vesicular throughout; action of the heart still much exaggerated ; impulse not strong as on the 20th; first sound feeble; still offers the bellows sound. The percussion over the heart is clear. The liver is not felt beyond the ribs.

*Nov.* 23*d.*—Respiration still completely absent at the lower part of the right side.

*Treatment.*—Took a laxative on the first day; then a solution of citrate of potassa and digitalis; afterwards the citrate was discontinued.

A case like the preceding might readily be mistaken for intermittent fever, on a superficial examination of the symptoms; but the history, as above detailed, clearly shows it to be altogether of a different nature. Nor is it hectic fever, to which it has many points of resemblance; for true hectic always depends upon a chronic irritation attended with a purulent discharge, and no such thing has occurred in the present case. It is the fever attending the commencement of acute tubercular disease, with inflammation of the pleura. The patient has pleurisy on both sides, which is an additional reason for believing it to be tuberculous. This form is to be distinguished from intermittent by the profuse sweats, pains in the chest, and other manifest signs of pulmonary disease; and by the absence of all signs of disease in the abdominal viscera. The difference between this fever and hectic has already been stated. Hectic fever attends no other lesion than confirmed phthisis, except large abscesses having a communication with the air; before they have been opened, the fever attending abscesses resembles intermittent, or rather that of commencing tuberculous disease.

*Of the lesions which occur in Intermittent Fever.*—At first there appears to be no material affection of any of the viscera ; the disease seems to spend its power upon the nervous system, and then upon the circulation. But in the progress of the disease, various organs become affected, and the blood is altered in its composition ; there is a deficiency of fibrine and colouring matter, and a redundancy of serum. The liver becomes enlarged, and in acute cases softened ; later in the disease it is apt to be hardened, and more or less cyrrhosed ; the spleen is still more enlarged, and is at first softened, then indurated. It is doubtful whether these lesions precede or follow the change in the constitution of the blood. This anemic condition is a point of great importance in the prognosis of intermittent fever, as it must be removed before we can consider the patient as cured ; the paroxysms may be arrested, but there is almost a certainty of their return, unless the healthy condition of the blood be restored. Hence we never wish to discharge a patient until his countenance resumes its natural appearance. In chronic intermittents, the heart is also affected ; there are palpitations, and a clear, clacking sound of the heart, as in the anemia, arising from other causes. The heart may also be inflamed in the more active form of the intermittent disease. In connexion with this affection of the heart, we often observe a neuralgic condition of that portion of the spinal marrow which corresponds to this organ, viz.: the upper dorsal vertebræ. There is pain in this region, varying in severity with the palpitations, and its removal must be effected, before the irregular action of the heart will cease. Besides this irritation of the spine, there are various other neuralgic affections consequent upon intermittent fever.

We come, lastly, to the consideration of a few points in the *treatment* of this disease. Cinchona or quinine is generally sufficient for its cure, and we therefore seldom give other remedies. This year we have given quinine in large doses, as five or six grains, repeated three or four times during the apyrexia. This method we find to be more effectual than our former practice of giving one or two grain doses frequently repeated. We have sometimes given as much as ten grains at a dose. After the paroxysms have been arrested, we reduce the amount to five grains daily. Towards the close of the disease, we put the patient on the use of iron, in order to restore the healthy condition of the blood.

During the present year I have been in the habit of combining rhubarb with quinine, in the proportion of one grain of the former to two of the latter, so that the rhubarb is in sufficient quantity to prove slightly laxative. This combination is peculiarly useful in the intermittents of children. We have not used mercurials this year, except as purges at the commencement of the disease. They act unfavourably in chronic cases of intermittent, where there is anemia, by diminishing the proportion of the solid elements of the blood, and thereby increasing the serum. In more acute cases, they may be employed to greater advantage.

In the few cases where bark and quinine have failed, we have used Fowler's solution with great success, in doses of ten drops, repeated three times a day. In one case, detailed above, it produced vomiting and cerebral symptoms, and the dose had to be reduced; but in the others no inconvenience resulted from its use. Arsenic should be resorted to, only when bark, as well as quinine, has failed; but the number of cases incurable by quinine even, will be found to be exceedingly small, if you give it in due proportion, and occasionally combine it with other remedies.

